# Dietary and physical activity habits of adults with inflammatory bowel disease in Aotearoa, New Zealand: A cross‐sectional study

**DOI:** 10.1111/1747-0080.70011

**Published:** 2025-04-07

**Authors:** Jia Min Yap, Catherine L. Wall, Kim Meredith‐Jones, Ella Iosua, Hamish Osborne, Michael Schultz

**Affiliations:** ^1^ Department of Medicine University of Otago Dunedin New Zealand; ^2^ Department of Medicine University of Otago Christchurch New Zealand; ^3^ Biostatistics Centre University of Otago Dunedin New Zealand; ^4^ Health New Zealand, Te Whatu Ora Southern Dunedin Public Hospital Dunedin New Zealand

**Keywords:** diet, inflammatory bowel disease, nutrition, physical activity

## Abstract

**Aims:**

To describe (1) dietary intake, food avoidance and adequacy, and (2) physical activity levels and barriers among New Zealand adults with inflammatory bowel disease.

**Methods:**

A cross‐sectional online survey comprising four questionnaires collecting data on demographics, disease activity index, dietary intake and physical activity levels was distributed. Exclusion criteria applied to those who were pregnant/lactating, with a stoma or pouch, or on enteral/parenteral nutrition. Descriptive analyses were performed, and dietary intakes were compared to established references. *T*‐tests, equality‐of‐medians tests and two‐sample proportion tests investigated differences between disease types.

**Results:**

Two hundred and thirteen adults with mostly quiescent or mildly active inflammatory bowel disease (53% Crohn's disease) completed at least one questionnaire. Participants were predominantly female (70%), New Zealand European (89%) with a median age of 37 years. Discretionary food intake was high, while fruit and vegetable consumption was generally suboptimal. Food avoidances were reported by 69% of participants, primarily dairy and vegetables. A higher proportion of participants with ulcerative colitis or inflammatory bowel disease‐unspecified avoided gluten and unprocessed red meat. Inadequate intakes of calcium (69%), selenium (40%) and magnesium (26%) were common. Most participants limited vigorous physical activity, but 67% met the physical activity guidelines. Barriers to physical activity were reported by 63% of participants, where fatigue (54%) and abdominal cramps (26%) were common barriers.

**Conclusion:**

Our findings demonstrate that New Zealand adults with inflammatory bowel disease had inadequate dietary intake and faced several barriers to physical activity, even when in remission.

## 1 INTRODUCTION

Inflammatory bowel diseases (IBD) are a group of chronic inflammatory conditions affecting mainly the gastrointestinal tract but often present with systemic manifestations.^1^ These diseases are characterised by a relapsing course with periods of active and quiescent disease. The two major subtypes are Crohn's disease and ulcerative colitis, while if the features do not clearly align with either subtype, IBD‐unspecified is diagnosed.^1^

Nutritional deficiencies are common in IBD as inflammation can damage the intestinal structure, resulting in nutrient malabsorption and enteric nutrient loss.^2^ Although protein‐energy malnutrition is more prevalent during active disease, micronutrient deficiencies can be present in quiescent phases, particularly in small bowel Crohn's disease.^3^ Factors such as poor appetite, diminished enjoyment of eating and self‐imposed food restrictions further increase risk of micronutrient deficiencies.^4^, ^5^ Studies showed that up to 66% of people with IBD modify their diets post diagnosis, and food restrictions may be more common in people with Crohn's disease than in those with ulcerative colitis.^6^, ^7^ Inconsistent dietary advice from healthcare providers may further contribute to dietary restrictions. Research indicates that 19% of IBD healthcare providers recommend avoiding or limiting fibre‐rich foods to prevent disease relapse,^4^ despite guidelines recommending a modified fibre diet only for symptomatic stricturing Crohn's disease.^3^ Additionally, the lack of guidance on re‐introducing fibre or dairy, which are often recommended to restrict to alleviate acute symptoms,^4^ can lead to prolonged restrictions during quiescent periods. Misinformation may also lead to unnecessary food restrictions,^8^, ^9^ negatively impacting nutritional adequacy in adults with IBD.

Physical activity may improve fatigue, quality of life and mental and physical health in IBD.^10^ However, physical inactivity is more common in this population compared to the general population, particularly in the initial stages of diagnosis.^10^, ^11^ Symptoms such as fatigue, abdominal cramps, gastrointestinal symptoms, muscle weakness, frequent medical procedures or hospitalisation can limit the ability to be active.^10^, ^12^ In some instances, people may intentionally refrain from doing physical activity for fear of worsening symptoms.

It is well known that a healthy diet and regular physical activity are key preventative measures against non‐communicable diseases,^13^ but this lifestyle may be challenging to implement for people with IBD.^2^, ^5^, ^8^, ^9^, ^10^, ^11^, ^12^, ^14^, ^15^, ^16^, ^17^, ^18^ To date, limited data exist on dietary and physical activity habits of people with IBD in New Zealand (NZ). This study aims to (1) assess dietary intakes, avoidances and inadequacies by comparing intakes to sex and age‐specific recommendations and (2) investigate usual physical activity levels and self‐reported barriers to physical activity among adults with IBD in NZ.

## 2 METHODS

This cross‐sectional study used an open survey with multiple questionnaires, available from 2021 to 2022. A convenience sampling method was used to recruit participants via the NZ patient charity Crohn's and Colitis NZ (~1500 patients registered) with an anticipated response rate of 10%–20%. Ethical approval was obtained from the University of Otago Human Ethics Committee (reference: H21/135), and the study followed the STROBE‐NUT framework^19^ and Checklist for Reporting Results of Internet E‐Surveys reporting guidelines.

Adults aged 18–80 years with IBD were eligible for the study, while those who were pregnant or lactating, had a stoma or pouch, or were on enteral or parenteral nutrition were excluded. A survey link containing study information, a consent form and survey content was distributed through the Crohn's and Colitis NZ newsletter and social media. Participants provided signed consent before participating in the study.

The REDCap web software^20^, ^21^ hosted four questionnaires: (1) demographics and health, (2) IBD disease activity index, (3) physical activity levels and (4) dietary intake. Most questions had formatted logic that required responses to proceed.

The demographics and health questionnaire collected data on age, gender (male, female and gender diverse) and ethnicity (NZ European, Māori, Samoan, Indian, Tongan, Niuean, Chinese, Cook Island Māori and others), smoking status, comorbidity/s, use of complementary and alternative medicines or supplements and IBD phenotype (Crohn's disease, ulcerative colitis or IBD‐unspecified), duration and medications. Participants could select multiple ethnicity options which were then grouped using the Ministry of Health classification system: Māori, other ethnicities and NZ European.^22^ This questionnaire took 5 min to complete.

Disease activity was self‐reported using the Harvey‐Bradshaw index for Crohn's disease and the simple clinical colitis activity index for ulcerative colitis and IBD‐unspecified.^23^ The Harvey‐Bradshaw index evaluates general well‐being, abdominal pain, number of liquid stools per day, abdominal mass and extraintestinal manifestations. The simple clinical colitis activity index assesses bowel frequency, urgency of defecation, blood in stool, general well‐being and extracolonic features.^23^ The disease activity score was calculated by summing the predefined scores of each item and categorised into the following: Harvey‐Bradshaw index scores: <5 remission, 5–7 mildly active and ≥8 active^24^ and simple clinical colitis activity index scores: ≤2 remission, <5 mildly active and ≥5 active.^25^ These questionnaires took <5 min to complete.

Physical activity levels were assessed with the validated modified Leisure‐Time Physical Activity Questionnaire tool.^26^ Participants reported time spent on mild, moderate and vigorous physical activities lasting at least 10 min over a typical week. The total minutes for each intensity level were summed over the past 7 days. Physical inactivity was defined as engaging in <150 min of moderate‐vigorous physical activity per week.^13^ A multiple‐choice question on perceived barriers to physical activity included options ‘fatigue,’ ‘muscle weakness,’ ‘abdominal pain,’ ‘joint pain,’ ‘bowel incontinence,’ ‘embarrassment related to symptoms,’ and ‘others,’ adapted from a previous NZ IBD cohort study.^12^ This questionnaire took <5 min to complete.

Dietary intake was assessed using the 2012 NZ Food Frequency Questionnaire (FFQ) long version,^27^ adapted to include recent food items based on the NZ Eating and Activity guidelines (2020)^13^ and supermarket inspections. These items included non‐dairy products, gluten‐free cereals, crackers and noodles, protein powder and takeaway meals. The nutrient profiles were sourced from FOODfiles 2016 or by entering individual foods into FoodWorks v.10.0 (Xyris Pty Ltd., Australia) to match nutritional labels, then incorporated into the original FFQ nutrient database (FOODfiles v.1.6.0, Way Down South Software, NZ). Participants estimated their consumption over the past 12 months, using a frequency scale of ‘Never or less than once a month,’ ‘1–3 per month,’ ‘1 per week,’ ‘2‐4 per week,’ ‘5‐6 per week,’ ‘1 everyday,’ ‘2–3 per day’ and ‘4–6 per day.’ The corresponding conversion factors were 0, 0.5, 1, 3, 5.5, 7, 17.5 and 35, respectively. Frequencies of intake were converted into macro‐ and micronutrient intake, and inadequate dietary micronutrient intake was assessed against the estimated average sex‐specific requirement references for Australia and NZ from the National Health and Medical Research Council.^28^ This questionnaire took 15 min to complete.

Food items were converted to align with the NZ healthy eating guideline portions^13^ and grouped into the following: (1) grains, (2) dairy and non‐dairy products, (3) fruits, (4) vegetables, (5) meat, poultry and fish, (6) legumes and lentils and (7) discretionary food and drinks. Discretionary foods were defined according to the Australian dietary guidelines as NZ has yet to provide a clear definition.^29^ These included confectionery, sugary, salty or high‐fat spreads and condiments, biscuits, muesli bars, commercial baking, processed meat, protein powder, sugar‐sweetened beverages and alcohol. Food avoidances were collected with a free text option and categorised by specific food components such as gluten‐containing foods, dairy, fruits, vegetables, red meat and fibre.^13^

Participants with at least one complete questionnaire were included in the analysis. Continuous variables were either presented as mean (SD) or median (lower quartile [LQ], upper quartile [UQ]) based on the data distribution. Categorical variables were presented as numbers (proportion). Previous research suggests dietary intake varies by IBD phenotype^6^, ^7^ and therefore, variables were stratified by IBD phenotypes (Crohn's disease and ulcerative colitis or IBD‐unspecified) and/or gender categories (male, female and other gender). Differences between IBD phenotypes for dietary intake, food avoidances, inadequate micronutrient intakes, physical activity levels and perceived barriers were investigated with a *T*‐test, equality‐of‐medians test and two‐sample proportion test depending on the outcome measure type and distribution. To investigate potential misreporting in dietary intake a sensitivity analysis was performed. This involved rerunning the primary analysis with removal of total energy intake outliers, defined as *z*‐scores below −3 or above 3.^30^

## 3 RESULTS

In total, 292 participants opened the survey link, 232 signed the consent form, 213 completed the demographics and health questionnaire, and 198 completed all four questionnaires after excluding two duplicates and one underage participant.

Participants were, on average, 37 years old (LQ, UQ: 26, 50), predominantly female (70.4%), and of NZ European descent (88.7%) (Table 1). Just over half were diagnosed with Crohn's disease while 46.5% of participants were diagnosed with ulcerative colitis or IBD‐unspecified with a median disease duration of 7 years (LQ, UQ: 2, 14). Most participants were in remission or had mildly active disease (70.3%) and were prescribed medications for their IBD (88.7%). Approximately one‐third of participants reported using complementary and alternative medicines or supplements including probiotics, curcumin, fish oil, vitamins and minerals. Thirty‐two participants (15.0%), mostly participants with Crohn's disease (*n* = 30), had previous surgical resections. A small proportion of participants (4.2%) were active smokers, and more than one‐third had one or more medical conditions other than IBD.

**TABLE 1:** Characteristics of participants with inflammatory bowel disease.

	Crohn's disease		Ulcerative colitis or IBD‐U		
	Male (*n* = 33)	Female (*n* = 81)	Male (*n* = 28)	Female (*n* = 69)	Other gender (*n* = 2)
Age, years, median (LQ, UQ)	33 (23, 51)	36 (24, 47)	37 (29, 51)	40 (26, 51)	36 (36, 36)
Ethnicity^a^, *n* (%)					
NZ European	30 (90.9)	74 (91.4)	25 (89.3)	59 (85.5)	1 (50.0)
Māori	3 (9.1)	2 (2.5)	0 (0)	2 (2.9)	0 (0)
Others	3 (9.1)	8 (9.9)	4 (14.3)	13 (18.8)	1 (50.0)
Smoking status, *n* (%)					
Never smoked	24 (72.7)	64 (79.0)	19 (67.9)	53 (76.8)	1 (50.0)
Active	1 (3.0)	3 (3.7)	5 (17.8)	0 (0)	0 (0)
Ex‐smokers	8 (24.3)	14 (17.3)	4 (14.3)	16 (23.2)	1 (50.0)
Comorbidities^b^—yes, *n* (%)	8 (24.2)	32 (39.5)	8 (28.6)	25 (36.2)	1 (50.0)
Years with IBD, median (LQ, UQ)	8 (5, 16)	7 (2, 16)	6 (2, 15)	5 (2, 12)	12 (5, 19)
Surgical resection—yes, *n* (%)	7 (21.2)	23 (28.4)	0 (0)	2 (2.9)	0 (0)
Prescribed IBD medication—yes, *n* (%)	29 (87.9)	73 (90.1)	20 (71.4)	65 (94.2)	2 (100.0)
Amino salicylates	2 (6.9)	14 (19.2)	10 (50.0)	38 (58.5)	1 (50.0)
Immunomodulators	16 (55.2)	44 (60.3)	2 (10.0)	34 (52.3)	1 (50.0)
Biologics	12 (41.4)	27 (37.0)	4 (20.0)	14 (21.5)	0 (0)
Corticosteroids	1 (3.5)	3 (4.1)	0 (0)	4 (6.2)	0 (0)
Complementary and alternative medicines or supplements—yes, *n* (%)	9 (27.3)	27 (33.3)	5 (17.9)	24 (34.8)	1 (50.0)
Disease activity^c^, *n* (%)					
Remission	15 (45.4)	23 (28.7)	8 (28.6)	16 (24.2)	1 (50.0)
Mild	12 (36.4)	25 (31.3)	13 (46.4)	33 (50.0)	1 (50.0)
Active	6 (18.2)	32 (40.0)	7 (25.0)	17 (25.8)	0 (0)
Number of missing observations	0	1	0	3	0

Abbreviations: IBD‐U, inflammatory bowel disease‐unspecified; LQ, lower quartile; UQ, upper quartile; *n*, number; NZ, New Zealand.

^a^ Self‐identified ethnicity was classified into three ethnic groups using the 2006 New Zealand census ethnicity questions and the Ministry of Health classification system. Participants could select multiple ethnicities, so column totals do not necessarily total 100%.

^b^ Self‐reported comorbidities were arthritis (*n* = 17), metabolic conditions (*n* = 13), liver and kidney conditions (*n* = 9), respiratory conditions (*n* = 14), heart conditions (*n* = 6), cancer (*n* = 6), skin conditions (*n* = 5), osteopenia/osteoporosis (*n* = 3), mental health conditions (*n* = 6) and others (*n* = 22).

^c^ Harvey Bradshaw Index (HBI) cut‐offs used for participants with Crohn's disease: remission <5, mildly active 5–7, active ≥8. Simple Clinical Colitis Activity Index (SCCAI) cut‐offs were used for participants with ulcerative colitis and IBD‐U: remission ≤2, mildly active <5, active ≥5.

Dietary habits results are presented in Table 2. The food category consumed most frequently was discretionary foods and drinks (5 serves/day; LQ, UQ: 3, 7) while legumes and lentils were the least consumed (0.4 serves/day; LQ, UQ: 0.1, 0.8). The consumption of these food categories did not differ between IBD phenotypes except for fruit intake, where those with Crohn's disease consumed significantly less compared to those with ulcerative colitis or IBD‐unspecified (*p* = 0.017). The macro‐ and micronutrient intakes were not significantly different between IBD phenotypes. Approximately two‐thirds of participants reported avoiding at least one food or drink, and there was no evidence that this was more common in one IBD group than another.

**TABLE 2:** Dietary intake of participants with inflammatory bowel disease.

	Crohn's disease (*n* = 105)	Ulcerative colitis and IBD‐U (*n* = 93)	*p* Value
Daily food servings, median (LQ, UQ)			
Grains	2.9 (1.9, 4.3)	3.0 (1.7, 4.3)	0.77
Dairy and non‐dairy products	1.8 (1.0, 3.1)	2.1 (1.0, 3.3)	0.29
Fruits	1.3 (0.6, 2.3)*	1.8 (0.9, 3.1)*	0.02
Vegetables	2.9 (2.1, 3.8)	3.1 (1.6, 4.2)	0.26
Meats, poultry and fish	1.0 (0.7, 1.4)	1.1 (0.7, 1.4)	0.28
Legumes and lentils	0.3 (0.1, 0.8)	0.4 (0.2, 0.9)	0.20
Discretionary food and drinks^a^	5.2 (3.4, 7.2)	4.9 (3.2, 6.1)	0.20
Daily intake, mean (SD)			
Energy (kJ)	9997.4 (4498.2)	10 178.8 (4870.8)	0.79
Carbohydrate (%TE)	43.3 (6.5)	43.4 (7.0)	0.91
Fat (%TE)	37.9 (5.0)	37.5 (5.6)	0.59
Mono‐unsaturated fat (%TE)	13.5 (2.4)	13.2 (2.5)	0.39
Poly‐unsaturated fat (%TE)	6.1 (1.6)	6.1 (1.5)	1.00
Saturated fat (%TE)	16.0 (2.9)	15.8 (3.5)	0.66
Protein (%TE)	17.7 (3.5)	17.4 (3.0)	0.52
Fibre (g)	29.0 (13.5)	31.8 (15.7)	0.18
Fibre (g)/4184 (kJ)	12.5 (3.3)	13.2 (3.0)	0.12
Alcohol^b^ (g)	1.4 (0.1, 5.2)	3.2 (0.1, 9.6)	0.09
Calcium (mg)	810.0 (428.4)	805.3 (409.9)	0.93
Iron (mg)	16.7 (8.2)	16.8 (8.7)	0.93
Zinc (mg)	13.6 (7.3)	13.3 (5.8)	0.75
Selenium (μg)	65.9 (38.8)	70.4 (39.8)	0.42
Thiamin (mg)	3.0 (2.9)	2.7 (2.8)	0.46
Riboflavin (mg)	3.2 (2.0)	2.9 (1.6)	0.25
Niacin (mg)	44.3 (22.2)	42.9 (19.5)	0.64
Vitamin B6 (mg)	2.3 (1.0)	2.5 (1.2)	0.20
Folate (μg)	589.0 (398.6)	561.3 (381.8)	0.61
Vitamin B12 (μg)	5.9 (4.1)	6.3 (4.9)	0.53
Food avoidance—yes, *n* (%)	70 (66.7)	66 (70.9)	0.52

Abbreviations: IBD‐U, inflammatory bowel diseases‐unspecified; kJ, kilojoule; LQ, lower quartile; *n*, number; SD, standard deviation; TE, total energy intake; UQ, upper quartile.

^a^ Discretionary foods and drinks include confectionery, sweet spreads (jam, syrups and honey), high‐fat spreads (pure butter and full‐fat mayonnaise), marmite, biscuits (sweet and savoury), muesli bars, baking (cakes and puddings) chocolate, savoury pies/pastries, processed meat, protein powder, sugar‐sweetened beverages (fizzy drinks, fruit juice and cordial), hot drinks (milo and chocolate) and alcoholic beverages.

^b^ Presented as median (lower quartile and upper quartile). All other values reported are mean (SD).

* *p* < 0.05 from *T*‐tests or equality‐of‐medians tests to determine differences for means and medians, respectively, between Crohn's disease and ulcerative colitis or inflammatory bowel disease–unspecified.

**FIGURE 1:**
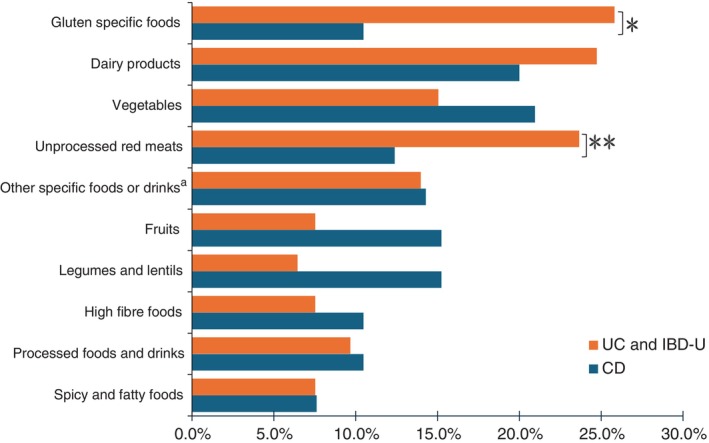
Percentage of participants with inflammatory bowel disease who avoided specific foods and drinks. *p‐value <0.05, **p‐value <0.005 for a difference in proportions test between Crohn's disease and ulcerative colitis or inflammatory bowel disease‐unspecified. ^a^Other specific foods and drinks include maple syrup/honey, white sugar, eggs, white rice and popcorn. CD, Crohn's disease; IBD‐U, Inflammatory bowel diseases‐unspecified, UC, ulcerative colitis.

Food avoidance was common in each IBD phenotype (Figure 1). A significantly higher proportion of those with ulcerative colitis and IBD‐unspecified avoided consuming gluten‐containing foods (difference: 0.15; 95% CI: 0.05, 0.26; *p* = 0.005) and unprocessed red meats (proportion difference: 0.11; 95% CI: 0.01, 0.22; *p* = 0.037) compared to those with Crohn's disease.

Table 3 compares the micronutrient intakes of our IBD population to recommended daily intakes. Inadequate calcium intake (68.9%) was most common, followed by low intakes of selenium (40.3%), magnesium (26.0%), folate (20.9%) and zinc (18.4%). The proportion of those with micronutrient intakes below the estimated average did not differ between IBD phenotypes. The sensitivity analysis identified six observations as outliers for total energy intake (mean: 26 993 kJ; SD: 1554 kJ). Removal of these outliers did not change the primary analysis inferences.

**TABLE 3:** Number (%) of participants with inflammatory bowel disease with inadequate dietary micronutrient intakes.

	Crohn's disease			Ulcerative colitis or IBD‐U		
	Male (*n* = 31)	Female (*n* = 74)	Total (*n* = 105)	Male (*n* = 28)	Female (*n* = 63)	Total (*n* = 91)
Vitamins^a^						
Thiamin (mg/day)	3 (9.7)	9 (12.2)	12 (11.4)	3 (10.7)	8 (12.7)	11 (12.1)
Riboflavin (mg/day)	2 (6.5)	3 (4.1)	5 (4.8)	2 (7.1)	3 (4.8)	5 (5.5)
Vitamin B6 (mg/day)	2 (6.5)	9 (12.2)	11 (10.5)	2 (7.1)	6 (9.5)	8 (8.8)
Folate (<320 μg/day)	7 (22.6)	15 (20.3)	22 (21.0)	3 (10.7)	16 (25.4)	19 (20.9)
Vitamin B12 (<2 μg/day)	2 (6.5)	4 (5.4)	6 (5.7)	3 (10.7)	7 (11.1)	10 (11.0)
Minerals^b^						
Calcium (mg/day)	21 (67.7)	53 (71.6)	74 (70.5)	16 (57.1)	45 (71.4)	61 (67.0)
Iron (mg/day)	0 (0)	7 (9.5)	7 (6.7)	1 (3.6)	5 (7.9)	6 (6.6)
Zinc (mg/day)	13 (41.9)	7 (9.5)	20 (19.0)	9 (32.1)	7 (11.1)	16 (17.6)
Magnesium (mg/day)	13 (41.9)	17 (23.0)	30 (28.6)	9 (32.1)	13 (20.6)	21 (23.1)
Selenium (μg/day)	14 (45.2)	33 (44.6)	47 (44.8)	13 (46.4)	19 (30.2)	32 (35.2)

*Note*: Two participants with ulcerative colitis identified as other genders were excluded from this analysis as estimated average requirements were sex‐specific. The difference in proportions tests was used to determine differences between Crohn's disease and ulcerative colitis or inflammatory bowel disease‐unspecified.

Abbreviation: IBD‐U, inflammatory bowel disease‐unspecified.

^a^ Inadequate intakes of vitamins are as follows: *Low thiamin intake* was defined as males: <1.0 mg/day; females: <0.9 mg/day. *Low riboflavin intake* was defined as males: <1.1 mg/day (18–70 years old), <1.3 mg/day (>70 years old); females: <0.9 mg/day (18–70 years old), <1.1 mg/day (>70 years old). *Low vitamin B6 intake* was defined as males: <1.1 mg/day (18–50 years old), <1.4 mg/day (>50 years old); females: <1.1 mg/day (18–50 years old), <1.3 mg/day (>50 years old).

^b^ Inadequate intakes of vitamins are as follows: *Low calcium intake* was defined as males: <840 mg/day (18–70 years old), <1100 mg/day (>70 years old); females: <840 mg/day (18–50 years old), <1100 mg/day (>50 years old). *Low iron intake* was defined as males: <6 mg/day; females: <8 mg/day (≤50 years old), <5 mg/day (>50 years old). *Low zinc intake* was defined as males: <12 mg/day; females: <6.5 mg/day. *Low magnesium intake* is defined as males: <330 mg/day (18–30 years old), <350 mg/day (>30 years old); females: <255 mg/day (18–30 years old), <265 mg/day (>30 years old). *Low selenium intake* is defined as males: <60 μg/day; females: <50 μg/day.

Table 4 presents physical activity levels and perceived barriers to physical activity. Two thirds of participants met the NZ physical activity guidelines (150 min/week of moderate‐vigorous physical activity) despite 62.9% reporting that IBD limited their physical activity levels. Participants reported spending more time each week carrying out mild (140 min; LQ, UQ: 60, 280) and moderate (140 min; LQ, UQ: 60, 300) intensity activities than vigorous intensity activities (33 min; LQ, UQ: 0, 140). Perceived barriers mostly included fatigue (54.0%), abdominal pain (25.7%), bowel incontinence (23.3%), joint pain (22.3%) and muscle weakness (20.3%). These physical activity levels and barriers also did not differ between IBD phenotypes.

**TABLE 4:** Physical activity levels and perceived barriers of participants with inflammatory bowel disease.

	Crohn's disease (*n* = 112)	Ulcerative colitis and IBD‐U (*n* = 94)	*p* Value^a^
Physical activity, median (LQ, UQ)			
Mild (min/week)	140 (60, 283)	140 (60, 280)	0.95
Moderate (min/week)	120 (60, 258)	150 (60, 360)	0.51
Vigorous (min/week)	20 (0, 108)	60 (0, 180)	0.12
Physical inactivity, <150 min of moderate‐vigorous per week, *n* (%)	44 (39.3)	25 (26.6)	0.05
Physical activity barriers^b^—yes, *n* (%)	69 (63.9)	58 (61.7)	0.74
Fatigue	60 (55.6)	49 (52.1)	0.62
Abdominal pain	25 (23.1)	27 (28.7)	0.36
Joint pain	29 (26.9)	16 (17.0)	0.09
Bowel incontinence	25 (23.1)	22 (23.4)	0.96
Muscle weakness	23 (21.3)	18 (19.1)	0.70
Bowel embarrassment	16 (14.8)	15 (16.0)	0.81

Abbreviations: IBD‐U, inflammatory bowel diseases‐unspecified; LQ, lower quartile; *n*, number; UQ, upper quartile.

^a^ *T*‐tests or equality‐of‐medians tests were used to determine differences in means or medians, respectively, between Crohn's disease and ulcerative colitis or inflammatory bowel disease‐unspecified.

^b^ Limitations to physical activity observations: Crohn's disease (*n* = 108), ulcerative colitis and IBD‐unspecified (*n* = 94).

## 4 DISCUSSION

Despite a plethora of literature showing that people with IBD often modify their lifestyle behaviours, inadvertently compromising nutritional adequacy and physical activity,^5^, ^7^, ^8^, ^9^, ^11^, ^12^, ^14^, ^15^, ^17^, ^31^, ^32^, ^33^, ^34^, ^35^, ^36^, ^37^, ^38^ very few studies have described these behaviours in a NZ population. We found that more than two‐thirds of the IBD cohort avoided foods, primarily dairy products and vegetables. Gluten‐containing foods and unprocessed red meats were more commonly avoided by people with ulcerative colitis. Intakes of discretionary foods and drinks were also high. These dietary habits may subsequently result in inadequate dietary intakes of calcium, selenium, magnesium, folate and zinc (in descending order), which were the most commonly identified in our study. Although more than half of this population met the NZ physical activity guidelines, barriers to physical activity such as fatigue, abdominal pain and bowel incontinence were common.

In our study, dairy products were among the most commonly avoided (22% of participants) food categories, which is consistent with previous literature that reports 12%–60% of people with quiescent IBD avoid dairy products.^9^, ^17^, ^31^, ^32^ Since dairy is the primary source of dietary calcium in NZ, avoiding these foods significantly restricts calcium intake. It was therefore unsurprising that low calcium intake was common (67.0%) although slightly less prevalent compared to other populations with IBD (72%–94%).^5^, ^15^, ^16^, ^17^ Calcium, along with vitamin D and phosphorus, is essential for skeletal health, and poor intake increases the risk of osteoporosis and fragility fractures.^28^, ^39^ The prevalence of osteoporosis in IBD is as high as 30%, and those with IBD are 40% more likely to develop osteoporosis compared to the general population.^39^ This increased risk is attributed to chronic inflammation, malabsorption, frequent use of corticosteroids, vitamin D deficiencies and likely inadequate dietary calcium.^1^, ^2^ Our findings support current recommendations to assess calcium intake in those with IBD, particularly in patients who exclude dairy products to help optimise calcium intake and support bone health.^3^

Consistent with previous literature,^7^, ^8^, ^9^, ^17^, ^32^, ^33^, ^34^, ^35^, ^36^, ^37^ several of the foods commonly avoided by our participants are high in dietary fibre, including vegetables, fruit, legumes and lentils, and gluten‐containing foods such as wheat, barley and rye. More participants with ulcerative colitis avoided gluten‐containing foods, which was consistent with one observational study^35^ while other studies have not reported differences in food avoidances between disease types.^7^, ^33^, ^34^ These discrepant findings may be due to cultural differences in food consumption, geographical locations and lack of standardised methods to measure food avoidance.^7^, ^33^, ^34^, ^35^, ^37^

Despite adults with IBD commonly reporting avoidance of high‐fibre foods, we found unexpectedly high intakes of fruits, vegetables and total fibre. For example, the average fruit consumption by those with ulcerative colitis was only just below the recommended 2+ serves/day,^13^ while those with Crohn's disease consumed 1.5 servings/day. Similarly, a meta‐analysis of patients in IBD remission reported an average fruit intake of 141 g (SD: 56), or roughly 1.5 serves/day.^40^ Vegetable intake was two servings less than the recommended 5+ servings/day, which is higher than other populations with quiescent IBD, where average intakes were 1–2 servings/day.^8^, ^40^, ^41^ Remarkably, our participants had an average fibre intake of 30.3 g/day (SD: 14.7), which contradicts existing literature that usually reports low fibre intake in those with IBD (9.9 ± 7.8 to 21.0 ± 10.5 g/day).^42^ Other studies report that high proportions (80%–91%) of those with IBD do not meet the national dietary fibre recommendations,^5^, ^41^, ^42^ compared to only 38% of our participants (analysis not shown). These varying findings are potentially due to differences in dietary data collection methods (food records vs. FFQ) and dietary fibre guidelines (ranging from 21 to 38 g/day). Recent data on fibre intake in the NZ general population is not available, but the NZ FFQ validation study reported a fibre intake 5 g/day higher than our cohort,^27^ as anticipated based on existing IBD literature.^40^ When fibre intake was expressed relative to total energy intake, our cohort still consumed slightly more fibre than a comparable cohort of people with inactive IBD, 12.8 g/4184 kJ (SD: 3.2) versus 10.5 g/4184 kJ (SD: 3.1).^5^ Nevertheless, high‐fibre foods were reported to be commonly avoided and one‐third of adults with predominantly quiescent or mildly active IBD still had inadequate fibre intake.

Avoidance of fibre‐rich foods by people with IBD is often linked to personal beliefs, misinformation from uncredible sources,^8^, ^9^ antiquated dietary advice from healthcare providers,^4^ or fear of functional gut symptoms, which coexist in 25%–30% of people with quiescent IBD,^43^ with increased intake. While removing these foods may provide temporary relief, prolonged avoidance may compromise micronutrient intake. For instance, a study found that people with IBD consuming fewer fermentable carbohydrates such as particular vegetables and fruits, legumes, lentils or wheat had significantly lower intakes of magnesium, zinc, folate and vitamin C than the general population.^5^ Thus, we speculate that avoiding these fibre‐rich foods, especially gluten‐containing foods like wheat flour or fortified cereals, may explain the inadequate dietary intakes of selenium, folate and magnesium in our study.^28^ Unlike findings from a meta‐analysis including people with quiescent IBD,^40^ we found comparable rates of inadequate micronutrient intake in both ulcerative colitis and Crohn's disease. Functional gut symptoms can affect both IBD phenotypes, potentially impacting micronutrient intake.^5^ Another possible explanation is that our study may have fewer participants with stricturing or penetrating Crohn's disease, which can also significantly affect dietary intake.^3^, ^6^

We also found a significantly higher proportion of those with ulcerative colitis than Crohn's disease who avoided unprocessed red meats. Despite epidemiological evidence linking red meat to worsening symptoms in ulcerative colitis,^44^ the literature surrounding red meat avoidance is conflicting, with some studies showing similar avoidance behaviours between disease types,^34^, ^35^ while others found that those with ulcerative colitis do not typically avoid red meat.^7^, ^33^ These inconsistent findings may reflect influences including cultural norms, animal welfare or health concerns, particularly colon cancer.^45^ Although reducing red meat intake is generally considered beneficial,^44^, ^45^ long‐term avoidance can lead to low intakes of highly bioavailable zinc and iron, both of which are common nutrient deficiencies in those with IBD.^2^ In contrast, plant‐based sources of zinc and iron are less easily absorbed due to the presence of phytates, although vitamin C from fruits and vegetables can improve absorption. However, these food sources of vitamin C are also often avoided by those with IBD. This highlights how multiple food restrictions can impact nutritional adequacy in this population.

The high intakes of discretionary foods and drinks in our study are an important finding as they often contain high amounts of added sugars, fat, alcohol and preservatives, which are common triggers for IBD symptoms.^7^, ^8^, ^9^, ^32^, ^34^ This finding is difficult to compare to other IBD populations as recent studies have mostly focused on specific discretionary foods or drinks such as sugar‐sweetened or carbonated beverages, rather than as a collective group.^14^, ^31^, ^41^, ^46^ Nonetheless, our participants consumed large amounts of discretionary foods and drinks, and 86% exceeded the Australian dietary guidelines for discretionary foods (males <3 serves/day and females <2.5 serves/day).^29^ A recent meta‐analysis showed that those with IBD in remission had excessive added sugar intake, indicating high consumption of discretionary foods.^40^ Such high intakes are worrying as discretionary foods and drinks have little nutritional value and likely displace nutritionally important foods that are commonly avoided in this population.

In line with other studies, many participants reported that IBD symptoms such as fatigue, gastrointestinal symptoms (abdominal pain and bowel incontinence), joint pain and muscle weakness were barriers to physical activity.^10^, ^12^, ^38^ Despite these challenges, 16% more of our participants met the physical activity guideline than the general population.^13^ This contrasts with previous research suggesting that physical inactivity is common among those with IBD.^10^ However, our results align with other studies showing similar physical activity levels in those with mostly quiescent IBD.^12^, ^47^ The differences in reported physical activity levels between studies are likely due to the varied methods used to collect physical activity data. Most NZ participants preferred mild or moderately intense activities over vigorous activity, consistent with previous studies demonstrating that walking is preferred over running or jogging for those with IBD.^12^, ^38^ Nonetheless, people with IBD should be encouraged to engage in regular physical activity of all modes, as there are reported benefits on quality of life, fatigue levels, mental health and physical health in those with IBD.^10^

Dietary intake and physical activity levels were only assessed once in this cross‐sectional study and therefore, seasonal variation cannot be determined. The results may also be prone to bias as they were self‐reported and we were unable to assess under‐ and over‐reporters due to the lack of objective measures. Our sensitivity analysis removing outliers for total energy intake, however, produced similar inferences. Furthermore, we used a NZ‐validated FFQ which reported a comparable average energy intake of approximately 9791 kJ, suggesting that our findings align with plausible estimates of energy intake within a NZ cohort. We also included physical activity examples from the NZ physical activity questionnaire to enhance familiarity and accuracy. Our participants were self‐selected and recruited through Crohn's and Colitis NZ and social media, and thus, our findings may not be generalisable to the wider IBD population. Notably, 70.4% of our participants were female and the majority were NZ European ethnicity. The prevalence of IBD in indigenous NZ Māori is low, but IBD usually affects males and females equally; therefore, the results may not represent the broader IBD population.^48^ Although we did not deliberately exclude people with active IBD, most participants in our study were in remission or had mildly active disease. For this reason, we were unable to assess the differences between disease activity for either dietary intake or physical activity.

Overall, our study found that most NZ adults with quiescent to mildly active IBD avoid certain foods and limit vigorous physical activity. The findings highlight that insufficient micronutrient intake, in particular calcium, magnesium, selenium, folate and zinc, may be related to food avoidance behaviours. Future efforts should focus on improving diet quality and understanding barriers and enablers to eating a balanced diet; thus, supporting health professionals to counsel patients and provide more patient‐centred care. Additionally, for those promoting physical activity, especially among patients not meeting the physical activity guidelines, assessing IBD‐related barriers and adopting a more personalised approach is recommended. Future research should also explore the feasibility of implementing these strategies with the IBD population.

## AUTHOR CONTRIBUTIONS

JMY, MS, CLW, KM‐J and HO contributed to the study concept and design. JMY and MS prepared the ethics application and carried out recruitment. JMY was responsible for data collection, data analysis and manuscript preparation. MS, CLW, KM‐J, HO and EI supervised and reviewed the content. JMY finalised the manuscript and all authors provided final approval. The authors acknowledge Professor Robin Turner for statistical guidance in the absence of Dr Ella Iosua.

## CONFLICT OF INTEREST STATEMENT

The author(s) have nothing to disclose.

## ETHICS STATEMENT

Ethical approval was obtained from the University of Otago Human Ethics Committee (reference: H21/135). All participants provided written informed consent prior to data collection.

## Data Availability

The data that support the findings of this study are available from the corresponding author upon reasonable request.
